# Exposure of Infants to Isoniazid via Breast Milk After Maternal Drug Intake of Recommended Doses Is Clinically Insignificant Irrespective of Metaboliser Status. A Physiologically-Based Pharmacokinetic (PBPK) Modelling Approach to Estimate Drug Exposure of Infants via Breast-Feeding

**DOI:** 10.3389/fphar.2019.00005

**Published:** 2019-01-22

**Authors:** Estella Dora Germaine Garessus, Hans Mielke, Ursula Gundert-Remy

**Affiliations:** ^1^Unit Epidemiology, Statistics and Mathematical Modelling, Department Exposure, German Federal Institute for Risk Assessment (BfR), Berlin, Germany; ^2^Institute for Clinical Pharmacology and Toxicology, Charité – Universitätsmedizin, Corporate Member of Freie Universität Berlin, Humboldt-Universität zu Berlin, Berlin, Germany; ^3^Berlin Institute of Health, Berlin, Germany

**Keywords:** breast milk, exposure, infants, isoniazid, PBPK, pharmacokinetics, tuberculosis

## Abstract

Isoniazid is a first-line anti-tuberculosis drug recommended for treatment of drug-susceptible *Mycobacterium tuberculosis* infections. Breast-feeding is not contra-indicated while undergoing isoniazid therapy, even though isoniazid was found to migrate into breast milk, leading to infant drug exposure. Exposure assessment of isoniazid in infants exposed to the drug via breast milk has so far not accounted for the polymorphic expression of the isoniazid metabolising enzyme *N*-acetyltransferase 2. The aim of this study was to re-visit the safety assessment of maternal isoniazid therapy for infants exposed to the drug via breast milk, while accounting for fast and slow metabolisers in the adult and infant population, as well as for slower metabolism in small infants than in adults. We applied a physiologically-based pharmacokinetic (PBPK) modelling approach to estimate mother and infant external and internal drug exposure non-invasively. Validity of our PBPK models was confirmed through comparison of simulated results with experimental data. Highest recommended oral doses for mothers are daily 300 mg or 900 mg every 3 days. Simulation of maternal intake of 300 mg resulted in oral exposures of 0.58 (95%CI: 0.42–0.69) mg/day and 1.49 (1.22–1.50) mg/day for infants of fast and slow metabolising mothers, respectively. Oral exposures of infants within the first 24 h after maternal intake of 900 mg were 1.75 (1.25–2.06) mg/day and 4.46 (4.00–4.50) mg/day. Maximal drug concentrations in infant plasma ranged between 0.04 and 0.78 mg/L for the two dosing regimens. We therefore conclude that infant exposure to isoniazid via breast milk after maternal drug intake of highest recommended doses is very low. We expect that such low exposure levels most likely do not cause any clinically significant adverse effects in nursed infants.

## Introduction

Tuberculosis is an infectious disease that is caused by *Mycobacterium (M.) tuberculosis* (World Health Organization [WHO], 2016). According to most recent data from the World Health Organization (WHO, data from 2015), the disease currently ranks as the 9th most common cause of death worldwide. In 2015, tuberculosis was reported as the cause of fatality in 1.4 million cases (World Health Organization [WHO], 2016). At present, around 2-3 billion people are infected with *Mycobacterium tuberculosis* (World Health Organization [WHO], 2017). Among female patients, reporting rates of tuberculosis are highest for women in the child-bearing age (Lawn et al., 2006). There is thus some probability that women are infected with *M. tuberculosis* while also being pregnant or breast-feeding their children. Pregnant and lactating patients suffering from drug-susceptible *M. tuberculosis* infections are advised to undergo drug treatment with first-line anti-tuberculosis drugs. Isoniazid, rifampicin, ethambutol, and pyrazinamide are the drugs recommended by several guidelines (Adhikari, 2009; Centers for Disease Control and Prevention [CDC], 2014; Mittal et al., 2014; Queensland Government. Department of Health, 2016).

According to the guidelines, breast-feeding is not contra-indicated while undergoing anti-tuberculosis therapy with isoniazid (and any other first-line anti-tuberculosis drug), even though isoniazid was found to migrate into breast milk (Lass and Bünger, 1953; Ricci and Copaitich, 1954; Berlin and Lee, 1979; Singh et al., 2007). The concentrations of isoniazid detected in breast milk were on such low levels, that drug exposure of the infant via breast milk was judged to be insignificant. Internal isoniazid exposure (i.e., plasma isoniazid concentrations) in infants exposed to the drug via breast-milk has so far only been measured once; the concentrations of isoniazid in plasma of breast-fed infants were found to be below the detection limit of 0.25 mg/L after maternal oral doses of 300 mg (Ricci and Copaitich, 1954). Due to ethical and practical constraints that hamper pharmacokinetic studies in infants, no further pharmacokinetic studies have been performed in infants exposed to isoniazid via breast-feeding.

Isoniazid is metabolised by the polymorphically expressed liver enzyme *N*-acetyltransferase 2 (NAT2), which divides the general adult and infant population into fast and slow metabolisers (Rey et al., 2001; Walker et al., 2009). Ricci and Copaitich (1954) did not measure the metaboliser status of their adult and infant study population. Therefore, isoniazid exposure of (slow metabolising) infants (of slow metabolising mothers) might have been underestimated. Hence, data on isoniazid plasma concentrations in slow metabolisers (infants and/or mothers) is highly warranted for proper safety assessment of breast-feeding for breast-fed infants of lactating mothers undergoing anti-tuberculosis therapy.

The aim of this study was to estimate non-invasively external (i.e., breast milk) and internal (i.e., plasma) isoniazid exposure in infants that are exposed to the drug via breast milk, while accounting for the polymorphic expression of NAT2, as well as for slower metabolism in young infants than in adults (Rey et al., 2001), in order to re-visit the safety of maternal isoniazid therapy for infants exposed to the drug via breast milk.

## Materials and Methods

### PBPK Model Development

#### Model Structure

**FIGURE 1:**
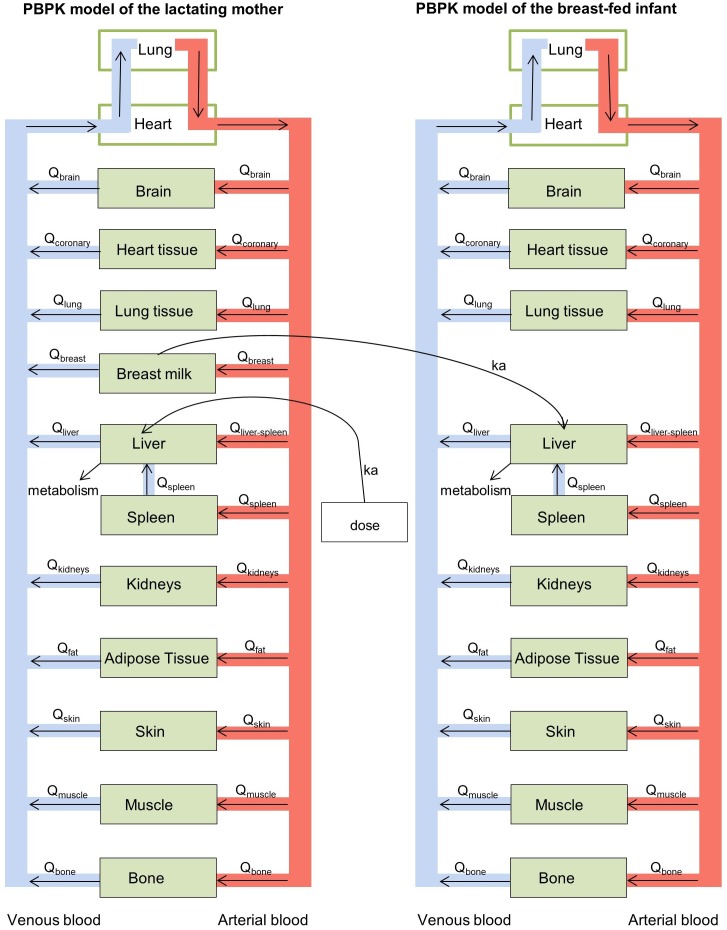
Schematic representation of the physiologically-based pharmacokinetic (PBPK) models for the lactating woman and breast-fed infant.

A physiologically-based pharmacokinetic (PBPK) modelling approach was used to estimate internal drug exposure in lactating mothers and external and internal exposure in breast-fed infants non-invasively. A PBPK model is a mathematical model that predicts the concentration-time profile of drugs in plasma and body organs, and mechanistically describes the processes (absorption, distribution, metabolism, and excretion) that guide the pharmacokinetics of drugs using both physiological parameters (e.g., organ volume and blood flows through the respective organs) and drug-specific parameters (e.g., partition coefficients and drug clearance rates). For the purpose of this study, two PBPK models were developed; one for a lactating mother and one for her infant. These two multi-compartment models were coupled to one another to allow for the prediction of internal drug exposure in the infant from drug intake by the mother. Each of the model compartments represented a body organ. All organs relevant for absorption, metabolism and excretion of isoniazid were included into the model. Additional organs were included into the model if organ weight data and data on blood flows through the organs were provided by the International Commission on Radiation Protection (ICRP) report of 2002 (ICRP, 2002) and if partition coefficients could be calculated with the data collection and relationships established by Schmitt (2008a,b). Breast milk was incorporated into the model as a separate compartment, receiving drug from blood directly, assuming breast milk blood flow to be identical to breast tissue blood flow. Figure 1 shows a schematic representation of the PBPK model structure for the lactating woman and her breast-fed infant.

#### Physiological Parameter Values

Organ weights, cardiac output and organ blood flows (*Q*_organ_) were taken from data presented in the ICRP report for adult women (ICRP, 2002). Data collected by the ICRP mainly describes the United States American population. In order to account for the generally leaner constitution of female tuberculosis patients, organ volumes (*V*_organ_) were re-calculated from organ masses assuming a density of 1 mg/L. Data on *V*_organ_ and cardiac output for newborns were taken from the same ICRP report. *Q*_organ_ are given as percentages of cardiac output. *Q*_organ_ in newborns were adjusted to the lower cardiac output (CO) of infants, the distribution of blood flows to the different organs (i.e., the percentages of CO) was, however, set to be identical to the one given for adult women. Breast milk volume was set to 0.1134 L (Kent et al., 2006).

#### Drug-Specific Parameters

Partition coefficients were calculated according to Schmitt (2008a,b). Physicochemical properties of isoniazid, required for the calculation of the partition coefficients, were retrieved from PubChem^1^; pKa: 1.82 (Perrin, 1965), logKow: -0.7 (Hansch and Hoekman, 1995), fraction unbound in plasma: 0.9 (Herrera et al., 1990), water volume/plasma volume: 0.935 (Beiling et al., 1966; Vitello et al., 2015). The partition coefficient for the breast milk compartment was taken from Singh et al. (2007), who measured an average (area under the isoniazid concentration-time curve- based) partition coefficient of 0.89 ([]_breastmilk_/[]_blood_). Table 1 lists the physiological and drug-specific parameters used in the two PBPK models.

**Table 1:** Physiological parameters and drug-specific parameters used in the two PBPK models.

Organ	*V* [L]		*Q* [L/h]		PC
			
	Mother	Infant	Mother	Infant	
Brain	1.3	0.38	42.48	4.32	0.73
Lung tissue	0.42	0.03	8.85^a^	0.9^a^	0.79
Heart tissue	0.25	0.02	17.7^b^	1.8^b^	0.70
Liver	1.4	0.13	95.58	9.72	0.70
Kidneys	0.275	0.025	60.18	6.12	0.74
Adipose tissue	22.5	0.93	30.09	3.06	0.15
Spleen	0.13	0.0095	10.62	1.08	0.75
Skin	2.3	0.175	17.7	1.8	0.62
Skeletal muscle	17.5	0.8	42.48	4.32	0.74
Bone	7.8	0.37	17.7	1.8	0.33
Breast milk	0.1134	–	1.416	–	0.89
Blood	3.9	0.27	–	–	–


*Chemical-specific model input parameters are: PC, ka, and Cl. Physiological model input parameters are: V and Q. ^*a*^Blood flow via bronchial arteries/veins. ^*b*^Blood flow via coronary arteries/veins. Cl, clearance; ka, absorption rate constant; PC, partition coefficient; Q, blood flow; V, volume.*

#### Absorption

Absorption of isoniazid after oral intake was characterised by a first-order absorption rate constant (ka) of 1.82 [/h] that was clinically determined (Wilikins et al., 2011) and was modelled via an absorption compartment. 100% of the dose was directed from the absorption compartment into the liver compartment, where (first-pass) metabolism took place. The mean bioavailability of isoniazid modelled using this technique was 86% (fast metabolising mother) or 93% (slow metabolising mother). The modelled bioavailability was thus comparable to the bioavailability of 78 and 93% observed by Saktiawati et al. (2016) for fed and fasted patients. In our model, in the absence of infant-specific data, ka was assumed to be identical for mothers and infants.

#### Distribution

Distribution of the drug was modelled assuming drug uptake into the organs to be limited by organ blood flow rate and organ capacity (i.e., organ volume and partition coefficient), not permeability, for simplicity reasons. Differential equations were used as described by Thompson and Beard for flow-limited PBPK models with well-stirred (organ-) compartments (Thompson and Beard, 2011) to model drug distribution:

(1) $$\frac{dA_{organ}}{dt}=\frac{A_{blood}}{V_{blood}}*Q_{organ}-\frac{\frac{A_{organ}}{V_{organ}}}{PC_{organ:blood}}*Q_{organ}$$

Where:

*A*_organ_, amount of drug in the organ
*A*_blood_, amount of drug in the blood
*V*_organ_, organ volume
*V*_blood_, blood volume
*Q*_organ_, blood flow to and from the organ
PC_organ:blood_, $\mathrm{partition}\;\mathrm{coefficient}\;=\;\frac{free\;drug\;concentration\;in\;the\;organ}{free\;drug\;concentration\;in\;blood}$ at equilibrium.

#### Metabolism and Excretion

Isoniazid is mainly metabolised by *N*-acetyltransferase 2 (NAT2) (Bonicke and Reif, 1953), an enzyme that is predominantly expressed in the liver (Bornschein, 2000; Windmill et al., 2000). NAT2 expression is polymorphic, splitting the general population into fast and slow (isoniazid-) metabolisers (Walker et al., 2009). In our model, in order to be able to take into consideration the effect of the metaboliser status (i.e., slow or fast metabolism) on the pharmacokinetics of isoniazid in the mother and in the infant population, elimination of isoniazid was described using two apparent isoniazid clearance values fitted from an adult population (Wilikins et al., 2011) and two apparent clearance values observed in 3 children aged less than 6 months (Rey et al., 2001). The clearance values (mean, 95% confidence interval) for the adult population were: 21.6 (18.9–28.2) L/h and 9.7 (9.61–10.7) L/h, for fast and slow metabolisers, respectively. In the infant population, the clearance values observed were: 2.55 (0.77–4.32) L/h and 0.76 (0.69–0.82) L/h in infants weighing 4 kg. In contrast to other PBPK models, this model did not restrict clearance to unbound drug molecules, as clearance estimates were based on “apparent” measurements that did not account for unbound drug fractions.

### Model Simulations

#### Mother–Child Combinations

Model simulations were done for the two NAT2 expression phenotypes in both the mother and infant population. We thus simulated four mother–child pairs: (a) mother: fast metaboliser – child: fast metaboliser, (b) mother: fast metaboliser – child: slow metaboliser, (c) mother: slow metaboliser – child: fast metaboliser and (d) mother: slow metaboliser – child: slow metaboliser.

In each simulation, we accounted for the variability of the mean clearance estimate. Simulation results are thus given as means with 95% confidence interval. The variability simulated thus represents variability in clearance estimates only.

#### Dosing Regimens for the Lactating Mother

Two dosing regimens for the mother were simulated:

Simulation 1: daily 300 mg isoniazid or
Simulation 2: 900 mg isoniazid two times a week (every 3rd day)

The two dosing regimens simulated represent the two maximal recommended doses of isoniazid in breast-feeding mothers (Centers for Disease Control and Prevention (CDC), 2016; Nahid et al., 2016).

#### Breast Milk-Drinking Behaviour of the Infant

Breast-feeding tuberculosis patients are advised to take in the drug immediately after a breast-feeding event (Jana et al., 1996, 2012). Assuming that a newborn drinks every 2 h, first drug intake would thus take place about 2 h after oral dosing of the mother. Therefore, milk drinking was modelled to take place every 2 h and for the first time 2 h post oral drug intake by the mother. It was simulated that at each drinking event, the infant would receive an oral isoniazid dose equal the total amount of drug molecules in breast milk at the time of drinking.

We used differential equations to describe the change of concentrations in the mother and infant over time. To simulate multiple-drinking by the infant (as well as to simulate multiple-dosing of the mother), we combined multiple tangens-hyperbolicus functions and thus obtained a smoothed version of a rectangular function which turns from 0 to 1 at pre-defined time points (namely 2 h, 4 h, 6 h, etc. post maternal drug intake); details are given in the supplemental material (Supplementary Material Section 1). By making use of an absorption compartment, which received the total amount of chemical present in breast milk at the time of drinking, drug uptake by the infant was modelled through multiplication of the present “dose” in the absorption compartment with the absorption rate constant ka.

#### Additional Simulations

The simulations (simulations 1 and 2) revealed that the time at which maximum isoniazid concentrations in breast milk are reached, are 0.8 h (fast metabolising mother) and 1.1 h (slow metabolising mother) post drug intake. In order to simulate maximal infant exposures, we performed additional simulations, in which the first time of drinking was set to the respective *t*_max_ times mentioned above (“simulation 3” and “simulation 4”). To simulate highest exposure and prevent underestimation of infant health risks, we also set the PC_milk:plasma_ to 1.42 (instead of 0.89), the highest reported individual isoniazid- milk:plasma ratio reported by Singh et al. (2007). The simulation results of these two simulations are presented in the Supplementary Material Section 2.

#### Relative Infant Dose Calculation

Relative infant dose (RID) was calculated with the following formula:

$$RID\;=\;\frac{Total\;oral\;dose\;of\;the\;child}{Oral\;dose\;of\;the\;mother}$$

Where:

Total daily oral dose of the child: sum of all (*n* = 12; drinking every 2 h in 24 h) drug amounts in breast milk at start of drinking event per day.

Relative infant doses can be used to estimate infant exposure via breast milk if internal drug exposure data from the breast-fed infant is not available.

### Model Validation

To validate the PBPK model of the mother, the model-predicted concentration-time profiles of isoniazid in plasma and breast milk were compared with experimental results of four clinical studies: (1) Lass and Bünger (1953), (2) Ricci and Copaitich (1954), (3) Berlin and Lee (1979), and (4) Singh et al. (2007). For comparison of simulated results with experimental results, simulated dosing regimens were matched to the clinical studies’ dosing regimens [(2), (3), (4): 300 mg/day, (1): 200 mg/day]. Validity was assessed by visual inspection.

For validation of the PBPK model of the breast-fed children, data from an experimental study, within which children aged between 0.13 and 196 months were exposed orally to isoniazid at doses of 10 mg/kg body weight (Rey et al., 2001), was compared visually with the simulated output (dosing: 10 mg/kg body weight, body weight: 4 kg).

### Sensitivity Analysis

A local sensitivity analysis was performed to investigate the model behaviour upon one-at-a-time changes in model input values.

### Software

Model simulations were performed in R version 3.4.1. (R Core Team, 2017), making use of the following R-packages: deSolve (for model simulations, calculation method applied: “lsoda”) (Soetaert et al., 2010), ggplot2 (for figure making) (Wickham, 2009) and zoo (for calculation of areas under the curves) (Zeileis and Grothendieck, 2005).

The model script including the model structure and differential equations has been added as a Supplementary Document to this manuscript.

## Results

### PBPK Model Validation

**FIGURE 2:**
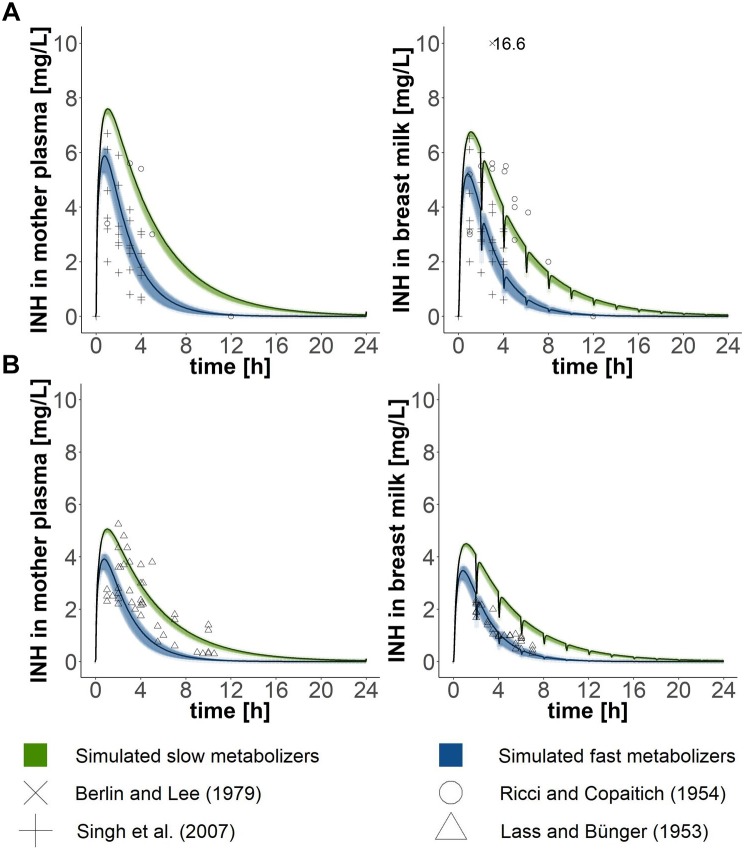
Validation of the PBPK model of the mother. Simulated concentration-time profiles (black lines) in (1) plasma and (2) breast milk were compared with data points from lactating mothers from four different clinical studies (Lass and Bünger, 1953; Ricci and Copaitich, 1954; Berlin and Lee, 1979; Singh et al., 2007), which either administered **(A)** 300 mg (Ricci and Copaitich, 1954; Berlin and Lee, 1979; Singh et al., 2007) or **(B)** 200 mg (Lass and Bünger, 1953) to mothers. Green and blue shadows show 95% confidence intervals of simulated mean estimates for slow and fast metabolisers, respectively.

**FIGURE 3:**
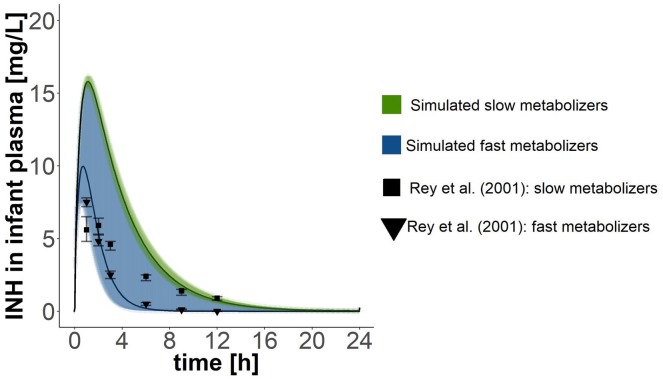
Validation of the PBPK model of the infant. Simulated pharmacokinetics after oral doses of 40 mg where compared with those reported by Rey et al. (2001), who administered 10 mg isoniazid/kg body weight (but did not report the weight of the children). Children in Rey et al. (2001)’s study where 42.5 months old on average (age range: 0.13–196 months). Points with error bars are means and standard errors of the respective means reported by Rey et al. (2001). Black lines show simulated mean concentration estimates with 95% confidence intervals.

Lactating women were exposed to an oral dose of 300 mg isoniazid in the studies by Ricci and Copaitich (1954), Berlin and Lee (1979), and Singh et al. (2007) and to 200 mg isoniazid in the study by Lass and Bünger (1953). When simulating the same exposure scenarios with our PBPK model for mothers, approximately two thirds of all data points collected within the experimental studies for isoniazid plasma concentrations lay within the simulated curves for fast and slow metabolising mothers (Figure 2). Isoniazid breast milk concentrations measured by Lass and Bünger (1953) matched well the isoniazid breast milk concentrations predicted with our PBPK model for fast metabolising mothers. Isoniazid breast milk concentrations of two women studied by Ricci and Copaitich (1954) were slightly underpredicted by our model. In contrast, some isoniazid concentrations in breast milk detected in women by Singh et al. (2007) were lower than the ones we predicted. For the most part, however, isoniazid concentrations in breast milk simulated were close to those observed in experimental studies by Lass and Bünger (1953), Ricci and Copaitich (1954) and Singh et al. (2007). Berlin and Lee (1979) reported one single concentration measurement of isoniazid in breast milk. The concentration measured by Berlin and Lee (1979) is much higher than the concentrations we simulated and those observed by Ricci and Copaitich (1954) and Singh et al. (2007). Plasma concentrations of isoniazid in fast and slow metabolising infants (Figure 3) overlapped due to the large confidence interval of the clearance estimate for infants that are fast metabolisers. Simulations of isoniazid concentrations in plasma of fast metabolising infants aged less than 6 months were almost identical to the ones that were observed by Rey et al. (2001). Isoniazid concentrations observed by Rey et al. (2001) for slow metabolising infants lay between the model-predicted concentrations for fast and slow metabolising infants.

### Pharmacokinetics of Isoniazid in Lactating Women and Breast-Fed Infants After Maternal Oral Drug Intake of Daily 300 mg

**FIGURE 4:**
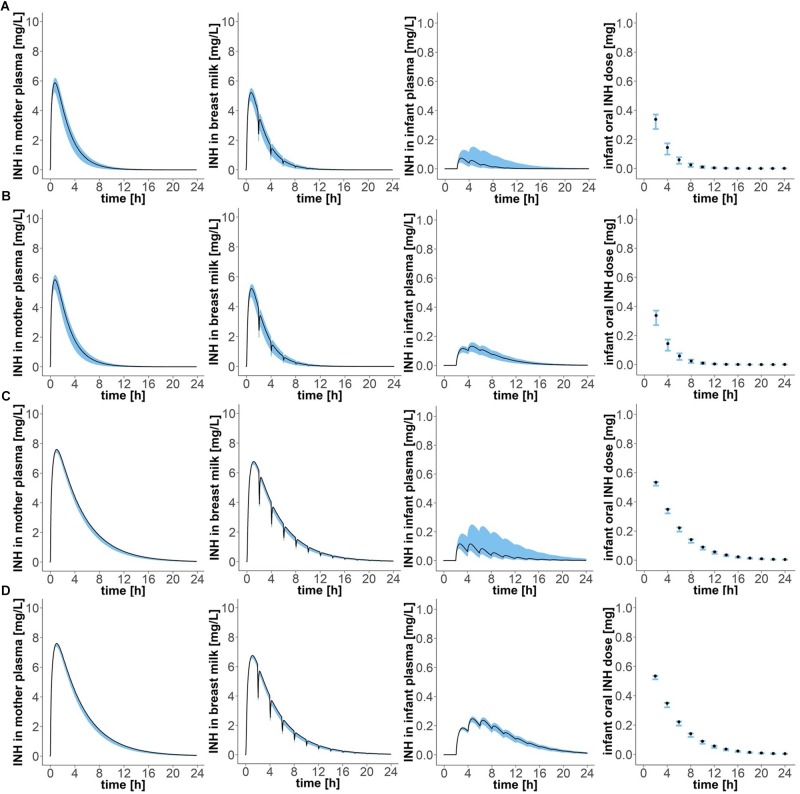
Concentration-time profiles of isoniazid in (1) plasma of the mother, (2) breast milk, (3) plasma of the infant, and (4) oral doses of the child, for four different mother–infant pairs; **(A)** mother fast – infant fast, **(B)** mother fast – infant slow, **(C)** mother slow – infant fast, **(D)** mother slow – infant slow. Simulation of maternal oral doses of daily 300 mg, breast-feeding every 2 h, the first breast-feeding event taking place 2 h after drug intake by the mother (“Simulation 1”). Black lines show simulated results for mean clearance estimates. Blue shadows show simulation results for all clearance values laying within the 95% confidence interval of the mean clearance estimate.

Simulation of oral administration of daily 300 mg to the mother and breast milk intake by the infant every 2 h, with the first event of drinking taking place 2 h after drug intake by the mother (“Simulation 1”), resulted in concentration-time profiles as depicted in Figure 4 and pharmacokinetic estimates as presented in Table 2. Maximal plasma concentrations were reached after 0.75 (0.65–0.80) h in fast metabolising mothers and after 1.05 (1.00–1.05) h in slow metabolising mothers. Maximal isoniazid plasma concentrations in lactating mothers were 5.88 (5.26–6.18) mg/L in fast metabolisers and 7.60 (7.40–7.62) mg/L in slow metabolisers. Isoniazid concentrations in breast milk were highest 0.8 (0.75–0.85) h or 1.1 (1.05–1.10) h after oral drug intake in fast or slow metabolising mothers, respectively, and the corresponding maximal isoniazid concentrations in breast milk were 5.22 (4.67–5.49) mg/L and 6.75 (6.58–6.77) mg/L. The resulting oral doses of breast-fed infants consequently were 0.58 (0.42–0.69) mg/day [relative infant dose: 0.2 (0.1–0.2) %] and 1.49 (1.22–1.50) mg/day [relative infant dose: 0.5 (0.4–0.5) %]. Consequently, maximal isoniazid concentrations in plasma were 0.07 (0.04–0.15) mg/L in fast metabolising infants of fast metabolising mothers, 0.13 (0.10–0.16) mg/L in slow metabolising infants of fast metabolising mothers, 0.12 (0.08–0.25) mg/L in fast metabolising infants of slow metabolising mothers and 0.25 (0.22–0.26) mg/L in slow metabolising infants of slow metabolising mothers.

**Table 2:** Pharmacokinetic parameter estimates of isoniazid after maternal oral dosing of daily 300 mg, breast-feeding every 2 h, the first breast-feeding event taking place 2 h after drug intake by the mother (“Simulation 1”).

Metaboliser status	Mother						Infant				RID [%]
			
	Breast milk			Plasma			Plasma			External exposure	
					
	*C*_max_ [mg/L]	*t*_max_ [h]	AUC [mg.24h/L]	*C*_max_ [mg/L]	*t*_max_ [h]	AUC [mg.24h/L]	*C*_max_ [mg/L]	*t*_max_ [h]	AUC [mg.24h/L]	Oral dose [mg/d]	
Mother f–infant f	5.22 (4.67–5.49)	0.80 (0.75–0.85)	17.32 (13.29–19.77)	5.88 (5.26–6.18)	0.75 (0.65–0.80)	19.80 (15.17–22.62)	0.07 (0.04–0.15)	2.70 (2.55–4.65)	0.28 (0.12–1.10)	0.58^a,c^ (0.42–0.69)	0.2(0.1–0.2)
Mother f–infant s							0.13 (0.10–0.16)	4.60 (4.50–4.65)	0.95 (0.63–1.23)		
Mother s–infant f	6.75 (6.58–6.77)	1.1 (1.05–1.10)	38.17 (34.69–38.51)	7.60 (7.40–7.62)	1.05 (1.00–1.05)	43.76 (39.76–44.16)	0.12 (0.08–0.25)	2.70 (2.55–4.75)	0.72 (0.38–2.39)	1.49^b,d^ (1.22–1.50)	0.5 (0.4–0.5)
Mother s–infant s							0.25 (0.22–0.26)	4.75 (4.70–4.75)	2.39 (2.00–2.65)		


*Numbers are means with 95% confidence intervals. Variability in mean estimates originates from variability in clearance estimates reported by Rey et al. (2001) and Wilikins et al. (2011). Simulations were done with mean, lowest limit and highest limit of the 95% confidence interval for the respective clearance values. ^*a*^2.00 (1.53–2.29) mg = isoniazid [mg] that is present in breast milk over 24 h (if the child did not drink anything during that time, AUC of mg for 24 h). ^*b*^4.44 (4.03–4.48) mg = isoniazid [mg] that is present in breast milk over 24 h (if the child did not drink anything during that time, AUC of mg for 24 h). ^*c*^0.15 (0.11–0.17) mg/kg bodyweight/day. ^*d*^0.37 (0.31–0.38) mg/kg bodyweight/day. AUC, area under the curve; f, fast metaboliser; RID, relative infant dose; s, slow metaboliser*.

### Pharmacokinetics of Isoniazid in Lactating Women and Breast-Fed Infants After Maternal Oral Drug Intake of 900 mg Every 3 Days

**FIGURE 5:**
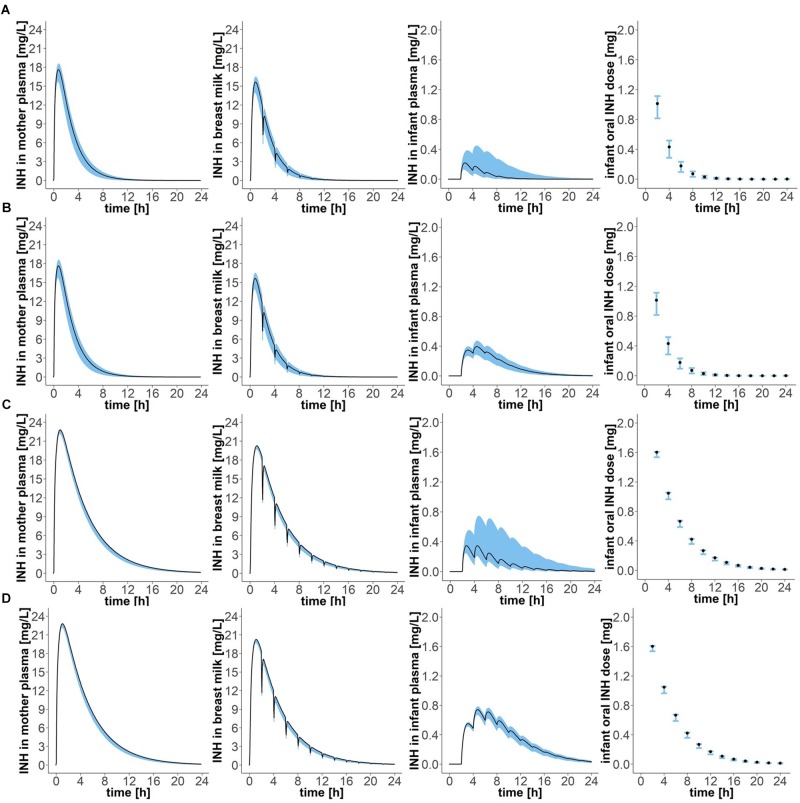
Concentration-time profiles of isoniazid in (1) plasma of the mother, (2) breast milk, (3) plasma of the infant, and (4) oral doses of the child, for four different mother-infant pairs; **(A)** mother fast – infant fast, **(B)** mother fast – infant slow, **(C)** mother slow – infant fast, **(D)** mother slow – infant slow. Simulation of maternal oral doses of 900 mg, breast-feeding every 2 h, the first breast-feeding event taking place 2 h after drug intake by the mother (“Simulation 2”). Black lines show simulated results for mean clearance estimates. Blue shadows show simulation results for all clearance values laying within the 95% confidence interval of the mean clearance estimate.

Oral intake of 900 mg every 3 days by lactating mothers resulted in maximal isoniazid concentrations in plasma of fast metabolising mothers of 17.63 (15.78–18.54) mg/L (Figure 5 and Table 3). Maximal isoniazid concentrations in slow metabolising mothers were 22.79 (22.21–22.85) mg/L. Maximal breast milk concentrations were 15.65 (14.00–16.46) mg/L and 20.26 (19.74–20.31) mg/L. As a result, infant oral doses were 1.75 (1.25–2.06) mg/day for infants of fast metabolising mothers or 4.46 (4.00–4.50) mg/day for infants of slow metabolising mothers. The relative infant doses calculated for this exposure scenario were consequently 0.6 (0.4–0.7) % and 1.5 (1.3–1.5) %. The corresponding maximal isoniazid plasma concentrations in infants were: 0.22 (0.13–0.45) mg/L (mother: fast – infant: fast), 0.40 (0.29–0.47) mg/L (mother: fast – infant: slow), 0.35 (0.25–0.74) mg/L (mother: slow – infant: fast) and 0.74 (0.67–0.78) mg/L (mother: slow – infant: slow).

**Table 3:** Pharmacokinetic parameter estimates of isoniazid after maternal oral dosing of 900 mg every 3 days, breast-feeding every 2 h, the first breast-feeding event taking place 2 h after drug intake by the mother (“Simulation 2”).

Metaboliser status	Mother						Infant				RID [%]
			
	Breast milk			Plasma			Plasma			External exposure	
					
	*C*_max_ [mg/L]	*t*_max_ [h]	AUC [mg.24h/L]	*C*_max_ [mg/L]	*t*_max_ [h]	AUC [mg.24h/L]	C_max_ [mg/L]	*t*_max_ [h]	AUC [mg.24h/L]	Oral dose [mg/d]	
Mother f–infant f	15.65 (14.00–16.46)	0.80 (0.75–0.85)	51.95 (39.86–59.32)	17.63 (15.78–18.54)	0.75 (0.65–0.80)	59.39 (45.51–67.86)	0.22 (0.13–0.45)	2.70 (2.55–4.65)	0.85 (0.36–3.31)	1.75^a,c^ (1.25–2.06)	0.6 (0.4–0.7)
Mother f–infant s							0.40 (0.29–0.47)	4.60 (4.50–4.65)	2.85 (1.89–3.68)		
Mother s–infant f	20.26 (19.74–20.31)	1.10 (1.05–1.10)	114.50 (104.07–115.54)	22.79 (22.21–22.85)	1.05 (1.00–1.05)	131.29 (119.29–132.48)	0.35 (0.25–0.74)	2.70 (2.55–4.75)	2.17 (1.15–7.16)	4.46^b,d^ (4.00–4.50)	1.5 (1.3–1.5)
Mother s–infant s							0.74 (0.67–0.78)	4.75 (4.70–4.75)	7.17 (6.00–7.95)		


*Numbers are means with 95% confidence intervals. Variability in mean estimates originates from variability in clearance estimates reported by Rey et al. (2001) and Wilikins et al. (2011). Simulations were done with mean, lowest limit and highest limit of the 95% confidence interval for the respective clearance values. ^*a*^6.01 (4.60–6.87) mg = isoniazid [mg] that is present in breast milk over 24 h (if the child did not drink anything during that time, AUC of mg for 24 h). ^*b*^13.31 (12.09–13.43) mg = isoniazid [mg] that is present in breast milk over 24 h (if the child did not drink anything during that time, AUC of mg for 24h).*^*c*^0.44 (0.31–0.52) mg/kg bodyweight/day. *^*d*^1.12 (1.00–1.13) mg/kg bodyweight/day. AUC, area under the curve; f, fast metaboliser; RID, relative infant dose; s, slow metaboliser*.

### Sensitivity Analysis

The local sensitivity analysis in the PBPK model for fast-metabolising mothers showed that the area under the curve of isoniazid concentration in plasma over time is most sensitive to 1% increases of the following five parameters (in decreasing order of sensitivity): clearance, PC_liver_, dose, *Q*_liver_ and *V*_breastmilk_. A 1% increase in mean clearance in the model for the fast metabolising mother resulted in a decrease of the area under the curve by 0.9988%. Similarly, an increase in PC_liver_ by 1% led to a decrease of the area under the curve by 0.9987%. An increase of 1% of the mean parameter value of dose, *Q*_liver_ and *V*_breastmilk_ caused the area under the curve- value to drop by 0.9889, 0.9866, and 0.0129%, respectively.

## Discussion

Isoniazid concentrations in plasma and breast milk of lactating mothers and/or plasma and urine of infants exposed to the drug via breast milk have been studied clinically in the past (Lass and Bünger, 1953; Ricci and Copaitich, 1954; Berlin and Lee, 1979; Singh et al., 2007). However, no study has previously estimated external and internal isoniazid exposure of infants via breast milk, while accounting for both polymorphic expression of NAT2, dividing mothers and infants into fast and slow metabolisers (Rey et al., 2001; Wilikins et al., 2011), and slower metabolism of isoniazid in infants aged less than 6 months than in adults, due to not yet mature expression of drug metabolising enzymes in infants (Rey et al., 2001). This study aimed at filling this research gap to re-visit the safety of breast-feeding during isoniazid therapy.

Our PBPK models simulated mother and infant plasma isoniazid concentrations that were in good agreement with plasma concentrations measured in patients. Similarly, simulated breast milk concentrations were generally comparable to the ones observed in patients, however, breast milk concentrations of 2 individuals of Ricci and Copaitich (1954) and the individual studied by Berlin and Lee (1979) were underestimated by 10 and 177%, respectively, and breast milk concentrations of three women in Singh et al. (2007)’s study were slightly overestimated by our model. Differences between the drug concentrations measured in the four experimental studies (Lass and Bünger, 1953; Ricci and Copaitich, 1954; Berlin and Lee, 1979; Singh et al., 2007) and the concentrations estimated in this study might be caused by (i) variability in body composition that is not captured by the model (e.g., variability in fat content, extent of expression and drug-affinity of drug transporters, pH in breast milk, etc.), (ii) variability in the time between birth and study start, which affects the composition of breast milk (and thus: partitioning of molecules into breast milk), the pH of breast milk (which may favour ion trapping) (Vorherr, 1974) and the pore size between epithelial cells covering the luminal surface of the breast milk compartment (which decreases with time after birth, restraining paracellular transport) (Anderson and Sauberan, 2016), or (iii) measurement/calculation errors. In our model simulations, we set the infant oral dose to the total amount of isoniazid in breast milk at the time of drinking in order to not underestimate infant drug exposure. Oral maternal doses of 300 mg each day or 900 mg every 3 days are the highest doses recommended by guidelines (Centers for Disease Control and Prevention (CDC), 2016; Nahid et al., 2016). After intake of 300 mg per os by the mother, maximal plasma concentrations in infants ranged between 0.07 (0.04–0.15) mg/L and 0.25 (0.22–0.26) mg/L. In the study by Ricci and Copaitich (1954), plasma concentrations of isoniazid in infants were below 0.25 mg/L after exposure via breast milk of mothers who were dosed 300 mg isoniazid orally. Hence, our modelling results are in good conformity with the experimental results.

Drug exposure of infants via breast milk was highest in slow metabolising infants of mothers with slow metaboliser status. Maternal doses of 900 mg isoniazid led to higher infant exposures than doses of 300 mg. Within the first 24 h after maternal intake of 300 mg isoniazid, external exposure of infants (weighing 4 kg) to isoniazid was 0.15 (0.11–0.17) mg/kg/day in infants of fast metabolising mothers and 0.37 (0.31–0.38) mg/kg/day in infants of slow metabolising mothers. Oral intake of 900 mg isoniazid by the fast-metabolising mother led to external infant exposure of 0.44 (0.31–0.52) mg/kg/day within the first 24 h after drug intake. Drug exposure of infants of slow-metabolising mothers taking a dose of 900 mg was 1.12 (1.00–1.13) mg/kg/day. The exposure of infants to isoniazid via breast milk after maternal drug intake of recommended doses is thus well below the recommended therapeutic dose of 10 mg/kg/day isoniazid for tuberculosis prophylaxis in infants (Mittal et al., 2014) and tuberculosis treatment in children with isoniazid (recommended infant dose range: 7–15 mg/kg/day, maximum dose: 300 mg/day) (World Health Organization [WHO], 2014).

Schaberg et al. (1996) studied the occurrence of adverse effects after treatment of (*n* = 519) hospitalised patients aged between 0.5 and 89 years suffering from pulmonary tuberculosis with daily doses of 5 mg isoniazid/kg bodyweight. Isoniazid treatment led to severe side effects (i.e., termination of drug treatment) in 7% of the study population. The most frequent adverse effect resulting from isoniazid treatment was found to be hepatotoxicity (occurrence in 4% of the population), followed by CNS toxicity (1.5%) and exanthema (1.2%). Risk factors for hepatotoxicity were found to be senior age (>60 years) and previous hepatitis (Schaberg et al., 1996). Liver toxicity due to isoniazid treatment has been related to isoniazid metabolite levels, particularly the levels of acetyl-isoniazid (Ac-INH) and isoniazid-hydrazine (INH-hz) (Cheng et al., 2013; Hassan et al., 2015). However, recent data point to the contribution of isoniazid itself, which is directly bioactivated (Metushi et al., 2016). A review by Steele and Burk (1991) of articles published between 1966 and 1989 reported incidences of hepatotoxicity caused by isoniazid monotherapy ranging between 0 and 2.9%. In (*n* = 232) children aged less than 15 years and treated for latent tuberculosis with isoniazid for 9 months at doses of 10 mg/kg body weight (maximal dose: 300 mg/day), 6.5% developed nausea and epigastric pain and 6% of all treatment-compliant children had elevated liver enzyme levels (Spyridis et al., 2007). These side effects were not judged to be serious by Spyridis et al. (2007). Similarly, in another study in which 506 children aged 2–17 years received isoniazid monotherapy for 9 months for treatment of latent tuberculosis infections (dose: 5 mg/kg bodyweight/day for children ≥12 years, 10 mg/kg bodyweight/day for younger patients) no severe adverse effect, either leading to death, life-threatening events, hospitalisation or irreversible damage was reported (Villarino et al., 2015). One child only (0.2%) developed a drug-related toxicity in that latter study (hepatomegaly and rash).

Since treatment of tuberculosis in children with isoniazid causes critical (hepato-) toxicity in less than 1% of all patients (Spyridis et al., 2007; Villarino et al., 2015) and as we estimated external infant exposure to isoniazid via breast milk after maternal drug intake of 300 mg every day or 900 mg every 3 days to be 10% or less than 10% of the oral dose recommended for treatment of tuberculosis in infants, we conclude that exposure of infants to isoniazid via breast milk will most likely not cause any clinically significant adverse side effects in infants. However, exposure to isoniazid via breast milk should nevertheless be accounted for in the dose selection if the infant itself needs to undergo anti-tuberculosis treatment. As peak plasma (and breast milk) concentrations in the mother occurred at roughly 1 h after drug intake in our simulation study [0.8 (0.75–0.85) h in fast metabolisers and 1.1 (1.05–1.1) h in slow metabolisers], and peak isoniazid concentrations in breast milk were observed in experimental studies after 1 h (Singh et al., 2007), 2 h (Lass and Bünger, 1953), 2 h or 3 h (Ricci and Copaitich, 1954) and 3 h (Berlin and Lee, 1979), we would like to further suggest mothers undergoing isoniazid monotherapy to breast-feed their infants at times where peak levels are not maximal (thus: before 0.8 h or after 3 h) in order to minimise infant drug exposure via breast milk.

Our study demonstrates the usefulness of PBPK models for the non-invasive prediction of drug exposure of infants via breast milk. Several PBPK models have previously been developed that aimed at describing the excretion of substances into breast milk in lactating women (Shelley et al., 1988; Byczkowski and Fisher, 1995; Byczkowski and Lipscomb, 2001; Verner et al., 2009; Partosch et al., 2018) or lactating rats (Byczkowski and Fisher, 1995; Clewell and Gearhart, 2002). The models vary in the way drug uptake into the breast milk compartment is described. While Verner et al. (2009) and Partosch et al. (2018) considered drug diffusion from plasma to take place via breast tissue only, Byczkowski and Fisher (1995), Byczkowski and Lipscomb (2001) assumed direct passage of substances from blood into breast milk and neglected drug uptake into breast milk via breast (-fat) tissue. Clewell and Gearhart (2002) proposed a PBPK model which accounts for both routes of drug transport into breast milk: transport via breast tissue and transport directly from blood. Our approach is thus similar to the one taken by Byczkowski and Fisher (1995), Byczkowski and Lipscomb (2001). The prediction of infant drug exposure via breast milk using a PBPK modelling approach is generally associated with uncertainties that may lead to inaccurate estimations of the pharmacokinetic characteristics of chemicals. For example, the prediction and description of physiological variability, the metabolism (especially in infants), chemical uptake and the apparent chemical permeability is still difficult. Based on our validation simulations, the model structure and parameter values chosen seemed accurate enough to mimic the physiological situation.

## Conclusion

This PBPK modelling study revealed that oral external exposures of infants to isoniazid via breast milk of mothers treated at recommended doses for tuberculosis infections is smaller or equal to 10% of doses recommended for tuberculosis treatment in infants. Consequently, drug exposure via breast milk after maternal drug intake of 300 mg each day or 900 mg every 3 days will most likely not result in clinically significant adverse side effects. The PBPK models developed within this study may be applied in future studies that aim to estimate drug exposure in infants via breast milk of mothers undergoing drug treatment.

## Other Information

A poster presentation on preliminary data of this study was presented at the 3rd German Pharm-Tox Summit that took place in Göttingen, Germany in February 2018. The corresponding abstract was published online (Garessus et al., 2018).

## Author Contributions

HM and UG-R formulated research question. EG, HM, and UG-R developed method and model. EG, HM, and UG-R analysed, interpreted results, and revised the manuscript. EG drafted the manuscript. All authors approved the final version of the manuscript.

## Conflict of Interest Statement

The authors declare that the research was conducted in the absence of any commercial or financial relationships that could be construed as a potential conflict of interest.

## Footnotes

1 https://pubchem.ncbi.nlm.nih.gov/
