# Supplementary material for: Exposure of Infants to Isoniazid via Breast Milk After Maternal Drug Intake of Recommended Doses Is Clinically Insignificant Irrespective of Metaboliser Status. A Physiologically-Based Pharmacokinetic (PBPK) Modelling Approach to Estimate Drug Exposure of Infants via Breast-Feeding
Source: Front Pharmacol. 2019 Jan 22;10:5. doi: 10.3389/fphar.2019.00005 (PMC6349757; doi:10.3389/fphar.2019.00005)
Supplement: Supplementary file 2 [file Data_Sheet_2.docx]

Supplementary Material

Exposure of infants to isoniazid via breast milk after maternal drug intake of recommended doses is clinically insignificant irrespective of metabolizer status. A physiologically-based pharmacokinetic (PBPK) modelling approach to estimate drug exposure of infants via breast-feeding.

Estella Dora Germaine Garessus^*^, Hans Mielke, Ursula Gundert-Remy^*^

*** Correspondence:** Corresponding Authors: estella.garessus@gmx.ch and ursula.gundert-remy@charite.de

# Table of contents

1. Modelling multiple dosing and multiple drinking (p.2)
2. Results of Simulation 3 and Simulation 4 (p.3)
3. References (p.4)
4. Supplemental Figures (p.5-6)
5. Supplemental Tables (p.7-9)
6. **Modelling multiple dosing and multiple drinking**

The differential equation solver deSolve (Soetaert and Petzoldt, 2010) was used in R version 3.4.1 (R Core Team, 2017) to simulate the pharmacokinetics of isoniazid. Multiple dosing of the mother and multiple drinking of the infant was modelled using a tangens hyperbolicus function. The tangens hyberbolicus function was written in such a way that solving of the equation resulted in an oral dose of 0 mg, unless the mother or infant took in drug or drank breast milk;

$$oral dose= dose*\frac{[\frac{tanh\left( 100*\left( t-t0 \right) \right)- tanh\left( 100*\left( t-t1 \right) \right)}{2}]}{{intake duration}_{drug or milk}}$$

Where:

t0: time at which dosing starts

t1: time at which dosing ends

intake duration_drug or milk_: t1-t0

The term in “[ ]” was added multiple times, to reflect multiple dosing (i.e. dose*([ ] + [ ] + []_n_)/duration drug or milk intake). The absorption compartment consequently had the following drug amount flow over time:

$$\frac{dA_{absoprtion comp.}}{dt}= -ka*A_{absoprtion comp.}+oral dose$$

Where:

A_absorption comp_: amount of drug in the absorption compartment

ka: absorption rate constant

Duration of drug intake was set to 0.0001 h (=0.36 sec) and duration of milk intake was set to 0.01 h (=36 sec).

1. **Results of simulation 3 and simulation 4**

In order to avoid underprediction of isoniazid exposure in infants out of precautionary considerations, additional simulations were performed, in which first time of breast-feeding was set to the time at which maximal isoniazid concentrations in breast milk are reached (0.8 h post drug intake in fast metabolizing mothers and 1.1 h post drug intake in slow metabolizing mothers). In addition, PCmilk:plasma was set to 1.42 (instead of 0.89), which is the highest reported milk:plasma- ratio of isoniazid in breast milk observed in an individual by Singh *et al.* (2007). Simulation 3 represents simulation of maternal oral doses of daily 300 mg, simulation 4 represents simulation of maternal oral doses of 900 mg every three days. Breast-feeding took place at a 2 h -interval. Results of Simulation 3 are shown in Figure S1 and Table S1. Results of Simulation 4 are shown in Figure S2 and Table S2.

1. **References**

R Core Team (2017). R: A language and environment for statistical computing. R Foundation for Statistical Computing, Vienna, Austria. *URL https://www.R-project.org/*

Rey, E., Gendrel, D., Treluyer, J.M., Tran, A., Pariente-Khayat, A., d'Athis, P., Pons, G. (2001). Isoniazid pharmacokinetics in children according to acetylator phenotype. *Fundamental & Clinical Pharmacology 15, pp.355-359.*

Soetaert, K., Petzoldt, T. (2010). Inverse Modelling, Sensitivity and Monte Carlo Analysis in R Using Package FME. *Journal of Statistical Software 33 (3), pp.1-28. URL:* [*https://www.jstatsoft.org/article/view/v033i03*](https://www.jstatsoft.org/article/view/v033i03)

Singh, N., Golani, A., Patel, Z. and Maitra, A. (2007). Transfer of isoniazid from circulation to breast milk in lactating women on chronic therapy for tuberculosis. *British Journal of Clinical Pharmacology 65 (3), pp.418-422*

Wilikins, J.J., Langdon, G., McIlleron, H., Pillai, G., Smith, P.J., Simonsson, U.S.H. (2011). Variability in the population of pharmacokinetics of isoniazid in South African tuberculosis patients. *British Journal of Clinical Pharmacology 21 (1), pp.51-62.*

1. **Supplementary Figures**

**
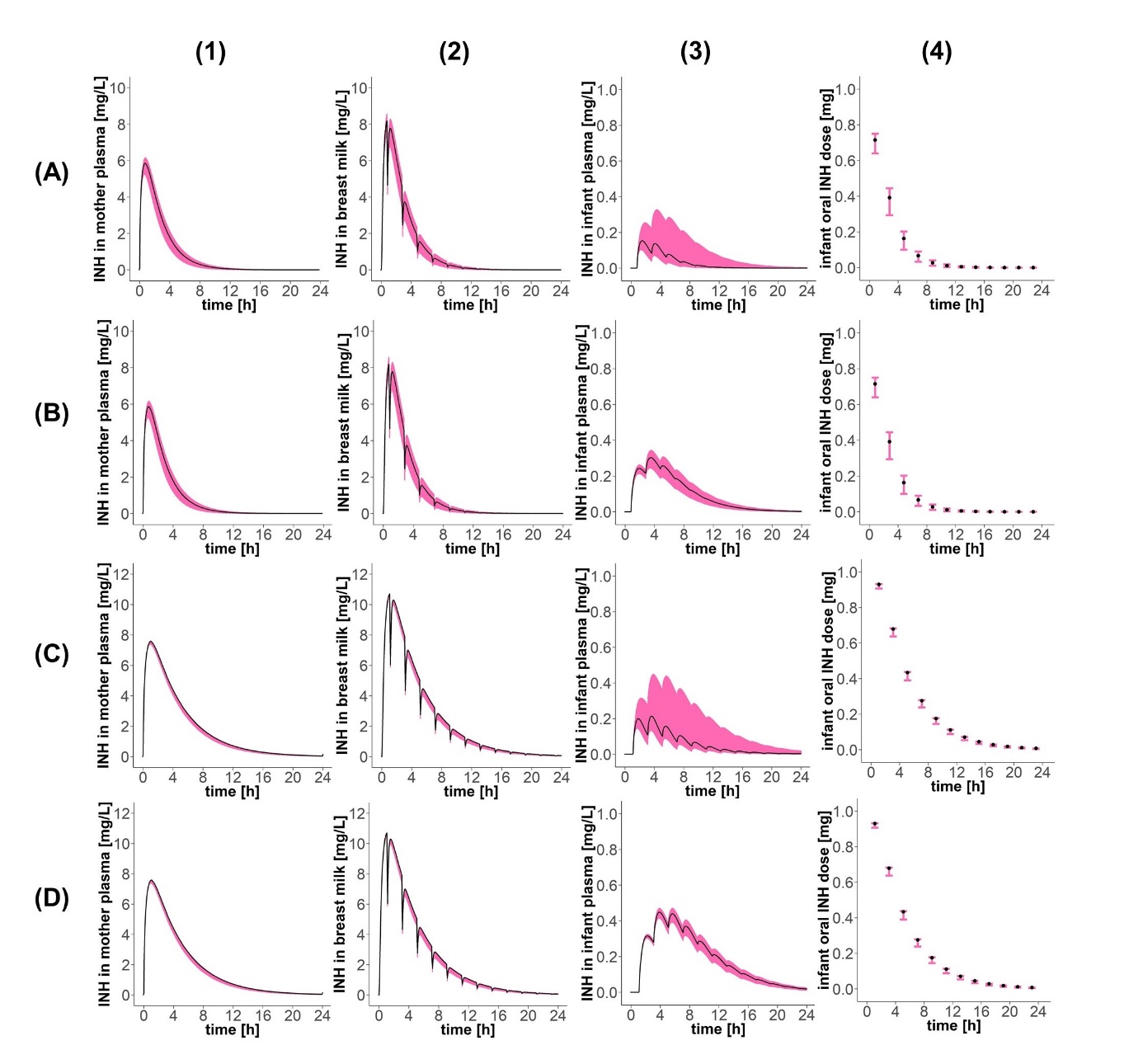
Figure S1.** Concentration-time profiles of isoniazid in (1) plasma of the mother, (2) breast milk, (3) plasma of the infant, and (4) oral doses of the child, for four different mother-infant pairs; (A) mother fast – infant fast, (B) mother fast – infant slow, (C) mother slow – infant fast, (D) mother slow – infant slow. Simulation of maternal oral doses of daily 300 mg isoniazid, breast-feeding taking place at an interval of 2 h, the first breast milk-drinking event taking place 0.8 h or 1.1 h after maternal drug intake, for infants of fast metabolizing mothers or slow metabolizing mothers, respectively (Simulation 3). PC_breast milk_ was set to 1.42, the highest reported individual concentration ratio of isoniazid in breast milk vs. plasma (Singh *et al.*, 2007). Black lines show simulation results of mean clearance values. Pink shadows show variability in simulation results as a result of variability in mean clearance estimates (i.e. lowest and highest limit of the 95% confidence interval of the mean estimate were simulated).


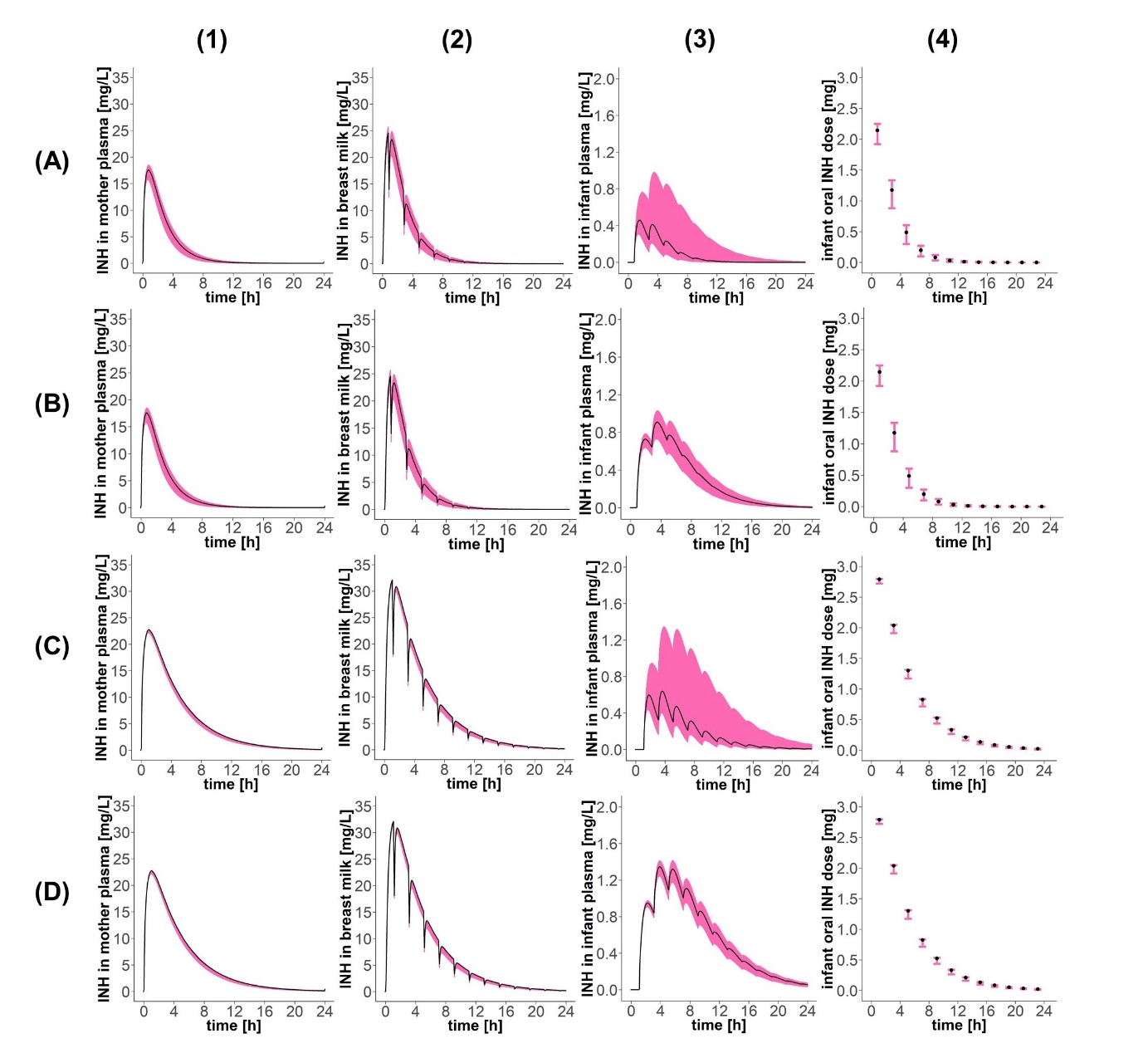
**Figure S2.** Concentration-time profiles of isoniazid in (1) plasma of the mother, (2) breast milk, (3) plasma of the infant, and (4) oral doses of the child, for four different mother-infant pairs; (A) mother fast – infant fast, (B) mother fast – infant slow, (C) mother slow – infant fast, (D) mother slow – infant slow. Simulation of maternal oral doses of 900 mg isoniazid every 3 days, breast-feeding taking place at an interval of 2 h, the first breast milk-drinking event taking place 0.8 h or 1.1 h after maternal drug intake, for infants of fast metabolizing mothers or slow metabolizing mothers, respectively (Simulation 4). PC_breast milk_ was set to 1.42, the highest reported individual concentration ratio of isoniazid in breast milk vs. plasma (Singh *et al.*, 2007). Black lines show simulation results of mean clearance values. Pink shadows show variability in simulation results as a result of variability in mean clearance estimates (i.e. lowest and highest limit of the 95% confidence interval of the mean estimate were simulated).

1. **Supplementary Tables**

**Table S1.** Pharmacokinetic parameter estimates of isoniazid after maternal oral dosing of daily 300 mg, breast-feeding every 2 h, the first breast-feeding event taking place at maximal breast milk concentrations (Simulation 3).

| **Metabolizer status** | **Mother** | | | | | | **Infant** | | | |
| --- | --- | --- | --- | --- | --- | --- | --- | --- | --- | --- |
|  | **Breast milk** | | | **Plasma** | | | **Plasma** | | | **External exposure** |
|  | **C_max_**  **[mg/L]** | **t_max_**  **[h]** | **AUC**  **[mg.24h/L]** | **C_max_**  **[mg/L]** | **t_max_**  **[h]** | **AUC**  **[mg.24h/L]** | **C_max_**  **[mg/L]** | **t_max_**  **[h]** | **AUC**  **[mg.24h/L]** | **Oral dose**  **[mg/d]** |
| **Mother f – infant f** | 8.19  (7.37-8.58) | 0.75  (0.75-0.75) | 26.88  (20.59-30.72) | 5.87  (5.25-6.17) | 0.75  (0.65-0.80) | 19.75  (15.14-22.55) | 0.15  (0.10-0.33) | 1.50  (1.35-3.50) | 0.67  (0.31-2.48) | 1.38^a,c,e^  (1.09-1.56) |
| **Mother f – infant s** |  |  |  |  |  |  | 0.30  (0.24-0.34) | 3.50  (3.40-3.55) | 2.23  (1.62-2.77) |  |
| **Mother s – infant f** | 10.70  (10.44-10.72) | 1.05  (1.05-1.05) | 59.55  (54.12-60.09) | 7.58  (7.39-7.60) | 1.05  (1.00-1.05) | 43.59  (39.62-43.99) | 0.21  (0.15-0.45) | 3.60  (1.65-3.85) | 1.34  (0.72-4.42) | 2.79^b,d,f^  (2.54-2.81) |
| **Mother s – infant s** |  |  |  |  |  |  | 0.45  (0.41-0.47) | 3.85  (3.85-5.65) | 4.44  (3.77-4.91) |  |
| Numbers are means with 95% confidence intervals. Variability in mean estimates originates from variability in clearance estimates reported by Wilikins *et al.* (2011) and Rey *et al*. (2001); Simulations were done with mean, lowest limit and highest limit of the 95% confidence interval for the respective clearance values.  ^a^3.19 (2.45-3.65) mg=isoniazid [mg] that is present in breastmilk over 24 h (if the child did not drink anything during that time, AUC of mg for 24h).  ^b^7.08 (6.43-7.14) mg=isoniazid [mg] that is present in breastmilk over 24 h (if the child did not drink anything during that time, AUC of mg for 24h).  ^c^0.35 (0.27-0.39) mg/kg bodyweight/day  ^d^0.70 (0.64-0.70) mg/kg bodyweight/day  ^e^RID [%]: 0.5 (0.4-0.5).  ^f^RID [%]: 0.9 (0.9-0.9)  Abbreviations: AUC: area under the curve, f: fast metabolizer, RID: relative infant dose, s: slow metabolizer | | | | | | | | | | |

**Table S2.** Pharmacokinetic parameter estimates of isoniazid after maternal oral dosing of 900 mg every 3 days, breast-feeding every 2 h, the first breast-feeding event taking place at maximal breast milk concentrations (Simulation 4).

| **Metabolizer status** | **Mother** | | | | | | **Infant** | | | |
| --- | --- | --- | --- | --- | --- | --- | --- | --- | --- | --- |
|  | **Breast milk** | | | **Plasma** | | | **Plasma** | | | **External exposure** |
|  | **C_max_**  **[mg/L]** | **t_max_**  **[h]** | **AUC**  **[mg.24h/L]** | **C_max_**  **[mg/L]** | **t_max_**  **[h]** | **AUC**  **[mg.24h/L]** | **C_max_**  **[mg/L]** | **t_max_**  **[h]** | **AUC**  **[mg.24h/L]** | **Oral dose**  **[mg/d]** |
| **Mother f – infant f** | 24.57  (22.10-25.73) | 0.75  (0.75-0.75) | 80.64  (61.76-92.15) | 17.60  (15.75-18.51) | 0.75  (0.65-0.80) | 59.24  (45.41-67.66) | 0.46  (0.31-0.98) | 1.50  (1.35-3.50) | 1.99  (0.93-7.45) | 4.15^a,c,e^  (3.26-4.68) |
| **Mother f – infant s** |  |  |  |  |  |  | 0.91  (0.73-1.03) | 3.50  (3.40-3.55) | 6.69  (4.87-8.30) |  |
| **Mother s – infant f** | 32.10  (31.32-32.17) | 1.05  (1.05-1.05) | 178.65  (162.36-180.27) | 22.75  (22.17-22.80) | 1.05  (1.00-1.05) | 130.77  (118.85-131.96) | 0.64  (0.44-1.34) | 3.60  (1.65-3.85) | 4.02  (2.16-13.27) | 8.36^b,d,f^  (7.61-8.43) |
| **Mother s – infant s** |  |  |  |  |  |  | 1.35  (1.24-1.41) | 3.85  (3.85-5.65) | 13.32  (11.3-15.74) |  |
| Numbers are means with 95% confidence intervals. Variability in mean estimates originates from variability in clearance estimates reported by Wilikins *et al.* (2011) and Rey *et al*. (2001); Simulations were done with mean, lowest limit and highest limit of the 95% confidence interval for the respective clearance values.  ^a^9.58 (7.34-10.95) mg=isoniazid [mg] that is present in breastmilk over 24 h (if the child did not drink anything during that time, AUC of mg for 24h).  ^b^21.24 (19.29-21.43) mg=isoniazid [mg] that is present in breastmilk over 24 h (if the child did not drink anything during that time, AUC of mg for 24h).  ^c^1.04 (0.81-1.17) mg/kg bodyweight/day  ^d^2.09 (1.90-2.11) mg/kg bodyweight/day  ^e^RID [%]: 1.4 (1.1-1.6)  ^f^RID [%]: 2.8 (2.5-2.8)  Abbreviations: AUC: area under the curve, f: fast metabolizer, RID: relative infant dose, s: slow metabolizer | | | | | | | | | | |
